# How Could Self-Determination Theory Be Useful for Facing Health Innovation Challenges?

**DOI:** 10.3389/fpsyg.2019.01870

**Published:** 2019-08-14

**Authors:** Laura Migliorini, Paola Cardinali, Nadia Rania

**Affiliations:** Department of Education Sciences, University of Genoa, Genoa, Italy

**Keywords:** health innovation, self-determination theory, childbirth plan, oncological genetic tests, health psychology

## Abstract

This paper offers a presentation of the characteristics of self-determination theory (SDT) in the health context as well as attempts to identify how this theory could be useful for facing health innovation challenges. Health innovation is based on scientific advances that have more complex relationships with health. This paper encourages the use of the SDT approach to face health innovation, both for physiological and pathological processes. In particular, the focus is on the changes and lifestyle choices related to physiological pregnancy and birth and to oncological genetic tests in the Italian context. The health innovation paradigm focuses on patients taking responsibility for making important health-related choices, and we think that SDT can offer new stimuli in light of the changes implemented from innovations in the field of health. The aim is that this manuscript will stimulate researchers to test the potential of this theory in the field of changing health-related processes. Practitioners are called upon to revise their orientation toward patients and, according to SDT, they should support autonomy rather than control the promotion of health-related change.

## Introduction

The concept of health for several decades has changed significantly with the overcoming of the biomedical model by the biopsychosocial model developed by Engel and colleagues (Engel, 1977; Schwartz and Weiss, 1978; Matarazzo, 1980; Schwartz, 1982; Kaplan, 1990; Anderson, 1998). According to Suls and Rothman (2004), the conceptual base for health psychologists is now the biopsychosocial model. This approach places the individual at the center of a large system influenced by multiple factors. In light of this more integrated approach, to understand both the well-being of the patient and the disease, it is necessary not only to worry about the body’s physical problems but also to focus on the psychological, relational and social aspects of the individual that are able to influence one’s well-being and/or malaise condition (Migliorini, 2015).

The World Health Organization defines health as the state of complete physical, mental, and social well-being and not merely as an absence of disease (WHO, 1948). The self-perception of health, characterized by positive emotional state toward oneself, a feeling of self-control and an optimistic view on the future, provides an important energy not only for coping with the difficulties of everyday life but also with those stressful conditions that threaten existence (Vazquez et al., 2009).

The need for greater attention to the psychological dimension within the potential of the biopsychosocial perspective may be met by advancing the theoretical basis through a rereading of SDT. In this manuscript, we consider the potential contribution of the SDT to the challenges that the health innovations paradigm poses to people’s well-being and malaise. In the last century, innovation had become a critical focus of all healthcare organizations (Lansisalmi et al., 2006). Innovative aspects such as new genetic test, digital information, or nanotechnology are revolutionizing health care, creating unexpected prospects for further innovation and improvement of existing processes (Omachonu and Einspruch, 2010). These changes aim at enhancing diagnostic and treatment options, life expectancy, quality of life, as well as the efficiency and cost effectiveness of the healthcare system (Varkey et al., 2008). Innovations in healthcare organizations are typically new services, new ways of working, generally more oriented to patient engagement, and/or new technologies, like the introduction of social media, of digital information, of more advanced diagnostic procedures (Lansisalmi et al., 2006). However, these scientific advances have made the relationship with health more complex. The process of innovation is both complex and multidimensional, and there are different key stakeholders involved, such as physicians and patients, who have unique and deliberate needs, wants and expectations. During the World Innovation Summit for Health (WISH) (2013) about patient engagement, empirical evidence of the benefits of patient engagement has been documented. Patient engagement is increasingly recognized as a core driver for the achievement of a high-performing health care system (Osborn and Squires, 2012). Engagement strategies improve patients’ health and reduce health care use and the costs associated with the risks of poor adherence, coordination of care failures, medical errors, and unnecessary emergency department visits and hospitalizations (Coulter, 2012).

This focus on patient engagement acknowledges that patients play an important role in their own health care. Furthermore, several studies have underlined the need to give attention to the well-being and to the satisfaction of patients during the care relationship (Rania et al., 2015, 2018). This includes patients working together with clinicians to discuss and select appropriate treatments or management possibilities. The health innovation paradigm developed and delivered new or improved health policies, systems, products and technologies that could improve people’s health. In particular, the focus of the present work is on the changes and lifestyle choices related to pregnancy and birth and to new diagnostic processes through genetic testing in the field oncology.

The health innovation paradigm introduces new scenarios not only from a medical point of view but also from a psychological one. The changes introduced by new technologies make the relationship with health more predictable but, at the same time, require a person to internalize the idea of inevitable risk (Battistuzzi et al., 2019). Enhanced accessibility to health related information (e.g., medical website, electronic recordkeeping that provide patients’ health history, magnetic resonance imaging, genetic testing, social media tools) can increase the perception of competence, but also the awareness of the potential risks for health and well-being associated to personal choices. Within these new perspectives, in which patients are inevitably faced with the advent of new technologies, new conceptual models are needed to grasp the change and the new styles of coping with the medical situations that are created. Although research applying the self-determination theory (SDT) to health care is present (Ryan et al., 2008; Ng et al., 2012; Kestler-Peleg et al., 2015), we think that the full potential of this theory is untapped. We think that the SDT can be a key to supporting these new processes involving patients on the one hand and the relationship with health care on the other.

## Self-Determination Theory

Self-determination theory (Ryan and Deci, 2000) underlines that individuals could be proactive or passive, according to the social conditions in which they are involved. Self-motivation could be fostered or inhibited by different situations. SDT differentiates two main types of motivation, according to the idea that “the type or quality of a person’s motivation would be more important than the total amount of motivation for predicting many important outcomes such as psychological health and well-being” (Deci and Ryan, 2008, p. 182). In particular, authors define autonomous versus controlled motivations. The first typology comprises both intrinsic motivation and the types of extrinsic motivation which people have identified with an activity’s value that they would ideally integrate into their sense of self. Controlled motivation, in contrast, consists of both external regulation and introjected regulation of some aspects such as avoidance of shame, contingent self-esteem, and ego-involvement.

Autonomous motivation tends to produce greater psychological health and more effective performance. Another important aspect of the theory concerns the recognition of psychological individual needs. According to Ryan and Deci (2000), there is a “set of universal needs that must be satisfied for effective functioning and psychological health” (p. 183); they argue that needs for competence, autonomy, and relatedness predict psychological well-being in all cultures (Deci and Ryan, 2008; Ohajunwa and Mji, 2018). Competence consists of feeling able to act on the environment and to reach a desired goal. Autonomy refers to the possibility of personally deciding what is best for oneself. The need for relatedness concerns the need to maintain and establish relationships in a social environment, for example, with health personnel. The vitality of basic psychological needs allows people to act more autonomously and to persist more at important activities (Ryan and Deci, 2017) like health-related choices both in physiological and pathological transitions. These needs become particularly salient when the individual experiences a strong experience like pregnancy and childbirth that, even if they are normative events, they require a reorganization of the self, based on these essential needs. Similarly, during a pathological transition, as genetic testing in the field oncology, the individual has to do relevant choices that drive the person to challenge him/her autonomy, competence and relatedness. People orient to the environment on the basis of information related to the initiation and regulation of behavior, so they experiment different causality orientations according to the extent to which they feel self-determined in general, in different situations and domains.

Causality orientation centered on autonomy has been positively related to psychological health and effective behavioral outcomes; a controlled orientation has been related to regulation through introjects and external contingencies, rigid functioning, and low well-being (Ryan and Deci, 2000); and “an impersonal orientation has been reliably associated with poor functioning and symptoms of ill-being, such as self-derogation and lack of vitality” (Deci and Ryan, 2008, p. 183). Intrinsic aspirations include such life goals as affiliation, generativity, and personal development, whereas extrinsic aspirations include such goals as wealth, fame, and attractiveness.

The autonomous orientation, in the interpretation of Deci and Ryan (1985), represents the highest degree of development, the maturity that allows one to autonomously adjust one’s behavior in harmony with the surrounding environment and to achieve good satisfaction in the interpersonal relationships, as well as a sense of self-realization. According to SDT, the pursuit of autonomous goals will improve well-being because these goals are in line with one’s true self, concerns, and values and therefore, satisfy the basic psychological needs. Conversely, the pursuit of controlled goals will thwart well-being because these goals do not accurately reflect the interests and values of one’s deeper self and are thus unlikely to satisfy basic psychological needs (Gillet et al., 2012).

**FIGURE 1:**
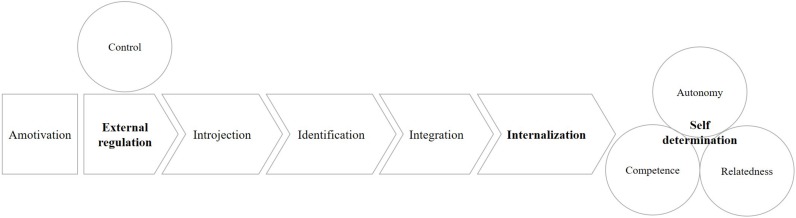
The SDT process of internalization.

Self-determination can be thought of as a continuum at the extremes of which we find intrinsic motivation and autonomous regulation on the one hand and the external determination of behavior and amotivation on the other (Figure 1).

Between the two poles, we can imagine a process of internalization of causality, through which the individual makes his own reasons for the behavior that others had initially presented to him.

There are therefore purely external forms of regulation (punishment and rewards) and more internalized extrinsic regulation forms, such as introjection, whose behaviors are guided by the dynamics of seeking approval, and identification, in which values are consciously accepted and transformed into elements of the self (Grolnick et al., 1991; Vallerand and Bisonette, 1992; Deci et al., 1994). Finally, the integration process organizes and makes congruent different identifications, making the experience of the self as a unit possible.

Internalization explains how values and self-regulations are transferred and maintained in one’s life, this process is very different than behaviorisms reinforcement which has no mechanism for transfer and maintenance of values and self-regulations. Literature underlines that being autonomous promotes internalization of values and awareness of intrapersonal and interpersonal dynamics and of their relation to behavior and health, in line with the psychosocial approach (Williams and Deci, 1996).

SDT has always maintained that the development of integrated, autonomous functioning depends on awareness. According to Deci and Ryan (2008), “mindfulness has been associated with autonomous motivation and with a variety of positive psychological and behavioral outcomes” p. 184. Miquelon and Vallerand (2008) show that having a higher sense of self-realization under challenge is consequential for health because it enables individuals to be protected against stressful events, as it provides them with sufficient psychological resources stemming from more adaptive forms of coping.

Research on SDT includes the development of many questionnaires, among which we can list the Intrinsic Motivation Inventory (Deci et al., 1994), the Aspirations Index (Kasser and Ryan, 1996), the Basic Psychological Needs Scales (Deci and Ryan, 2000), and the General Causality Orientations Scale (Deci and Ryan, 1985) that assess different constructs contained within the theory, as described above.

SDT is characterized by the concept of autonomous self-regulation, and autonomy should be considered an ethical mandate for medicine. Much of the research guided by SDT has also examined environmental factors that hinder or undermine self-motivation, social functioning and personal well-being. Some research applying SDT to health care exists (Ng et al., 2012), and we propose that this theory can offer new stimuli in light of changes implemented from innovations in the field of health. This paper encourages the use of the SDT approach to face health innovation both in physiological and pathological processes, by analyzing two exemplifying case studies of health innovation in the Italian context: the introduction of the Childbirth Plan and the BRCA test.

## Pregnancy and Birth

Childbirth can be considered an important ritual in the transition to parenting (Migliorini et al., 2016; Rania, 2019). Physiological pregnancy and birth also represent a significant context for the elements of the innovation process. For this reason, it is necessary to understand more deeply the psychological processes that are associated with the introduction of innovative patient-centered tools, in a physiological process such as birth without obstetric risk. Literature underlines that social media and mobile phone apps are increasingly popular among pregnant women; these innovative tools allow people to access health knowledge and learn to identify risk behaviors and warning signals during pregnancy (Chan and Chen, 2019). Giving information about maternal and infant care via the mobile phone app could support the need of competence and confidence in gaining the new role as a mother. Recent study (Backer and Yang, 2018) evidences on the use of social media as a source of information and social support for perinatal women; this could represent a way of adjusting to their new role, but also a way to satisfy the need of relatedness to other in this specific period.

The Childbirth Plan (Moore et al., 1995; Whitford and Hillan, 1998) is “a written communication tool prepared by a pregnant woman, which involves her preferences for the management of her labor and delivery” (Farahat et al., 2015; p. 24). It could be considered a tool of health innovation in Italy where its completion has been recently inserted in national guidelines [Italian Ministry of Health (2014–2018)], however, it has been offered only to 33.7% of women in North and to 14% of women in South (Mascolo and Demartis, 2017). Through this document, women were encouraged to clarify their needs, expectations, and preferences for the birth and to open up channels of discussion with their health-care providers. This allows expectant women to participate in the decision-making process for their own care during the birth process (Westergren et al., 2019). The engagement of women in prenatal discussions could increase their knowledge of obstetric healthcare and could help them better understand how to converse with health-care workers. Such discussions within the framework of Childbirth Plans thus improve self-confidence and positive birth experiences (Goodman et al., 2004).

According to Kuo et al. (2010) Childbirth Plans increase women’s sense of competence over the childbirth process, as well as their positive birth experiences. This plan represents for women an intervention that increases the feelings of confidence and the development of reasonable expectations toward future uncertainties. The Childbirth Plan gives back to women their important role in their own health care and supports the process of introjection and identification toward a causality orientation centered on autonomy and integration.

Women’s childbirth experiences are influenced by their perception to have some desired behavior during the childbirth process, as evidenced by their assessment of their ability to maintain their freedom and confidence during this process (Gibbins and Thomson, 2001; Cheung et al., 2007). High ratings of possibility to accomplish one’s desired goals have been shown to correspond to high satisfaction with the childbirth experience (Lavender et al., 1999; Cheung et al., 2007). The literature (Morano et al., 2018) underlines an extraordinary variety of feelings and emotions, which enrich, but may also complicate, life in the delivery room. Ambivalent emotions can be amplified by changes that can occur during childbirth. When a woman expects to have a normal vaginal birth but, for unexpected reasons, requires a cesarean birth, a positive relationship with health-care providers and an active involvement in decision making can outcome in feelings of satisfaction with the childbirth experience (Kuo et al., 2010). The Childbirth Plan could represent a significant tool to facilitate this process. However, some studies (Davis et al., 2019) have warned about the fact that “maternal actions that modify standard care processes have the potential for harm, without medical benefit” (p. 217). Therefore, the Childbirth Plan can be a useful tool, but if not structured within a woman/obstetric/medical alliance, it can become a means of detachment and mistrust.

In Italy, the law on “Protection of the rights of the pregnant woman, the promotion of physiological birth and the protection of newborn health” in 2006 proposes to promote greater protection of the rights of the pregnant woman and the newborn, and appropriate assistance from the National Health Service for the entire process of birth. In Law 219 of 2017, it is written “The time of communication between doctor and patient constitutes time of cure”. Communication is considered a medical act, as included in the WHO intrapartum care recommendations (WHO, 2018). The application of these principles to the Childbirth Plan means using a tool that collects and elaborates scientific knowledge, combines it with welfare behaviors that should be respectful of the woman’s emotional states and aims to maintain the maximum well-being of the parturient and the child.

Understanding and supporting the woman’s Childbirth Plan will allow supporting the woman’s autonomy and will likely lead to greater patient satisfaction with the birth process (Anderson and Kilpatrick, 2012). For patients, a Childbirth Plan is drawn up, in which the woman explains how she would like to give birth and what she would not like to do during labor and delivery (an episiotomy or Kristeller’s maneuver, for example), as well as her own postpartum and neonatal preferences, can be a means to repossess decisions about birth when possible in the absence of emergencies.

Planning birth is a part of prenatal care, and the use of innovation like an app interface could support flexibility in the user’s answers and any subsequent modifications made before delivery, this tool could empower women to be the protagonist during childbirth, promoting autonomy. The introduction of an in-app childbirth plan could be helpful also because it allows the access to health data and information on the smartphone or to transfer it from the app to local information systems (Moraes et al., 2019).

For clinicians, knowing the expectations, the anxieties, and the fears of the woman can help to increase empathy, enhance the woman/obstetric/medical alliance and fill the gap in care between the person who assists the woman during pregnancy and the one who assists her at birth. This perspective might help to reframe communication and planning as “a commitment by medical and allied health professionals to patient-centered innovation” (Batayeh et al., 2018; p. 2).

Considering the physiological experience of childbirth, we can say, for example, that the indications for doctors and obstetricians to the use the epidural, if not previously planned, can limit the sense of autonomy of women; according to Ryan and Deci (2000), pressured evaluation and imposed goals could decrease intrinsic motivations. The pursuit of controlled goals will obstruct well-being because these goals do not accurately reflect the interests and values of one’s deeper self. If the woman lives a situation of free choice, she maintains or increases the motivation for the task; instead, if she feels that the actions are imposed from the outside, she will feel less self-determined and intrinsically less motivated.

Within the decision-making processes concerning childbirth, the choice of a vaginal birth after caesarean (VBAC) deserves a special consideration. Some studies (Di Matteo et al., 1996; Fisher et al., 2006) underline the proliferation of cesarean sections and describe some possible predisposing factors to this trend: fear of labor pain, fears of malpractice litigation, clinician’s preference, practice patterns, and financial incentives for hospitals and physicians. Another possible explanation for the high rate of cesarean sections is the common belief that after a cesarean, a woman should continue the same method of delivery and that it is not possible for a following childbirth to be performed vaginally (Godden et al., 2012). These elements could represent external factors that could influence women’s motivation. The values and social context of pregnant women, positive relationships with midwives and the support from an informal network represent important factors involved in the choice of childbirth mode (Emmett et al., 2006; van den Berg et al., 2008; Kingdon et al., 2009; Klein, 2012).

For women, being part of the decision-making process increases trust in clinicians and gives a feeling of activation/implication and sense of freedom and confidence; however, this decision is difficult because there is limited information, and some clinicians consider VBAC a dangerous choice. According to Lundgren et al. (2012), choosing a VBAC is a strong personal responsibility for women; however, autonomous motivation could allow women to persist in their wish. Additionally, in this particular situation, the Childbirth Plan could be a useful tool to increase the sense of autonomy of women and to support their decision. Therefore, the basis of a self-determined conduct is the need to feel the authors of their actions and to freely choose the type of task and its mode of performance.

## Oncology and Genetic Testing

If it seems more physiologically plausible that a subject can freely choose the actions to be undertaken and can feel autonomous and responsible for one’s own decisions, the process of self-determination may appear more complex when one is faced with serious pathology. In the face of serious pathologies, such as cancer, the person has a difficult experience since living with cancer is associated with our finiteness and imminent death (Cavanna and Migliorini, 2015). According to biopsychosocial model, the literature emphasizes the importance of the holistic vision of the person in the oncology field (Cavanna et al., 2015). Considering the burden that breast cancer poses to the health system and the affected person, national and international communities are stepping up efforts to implement appropriate prevention and care for affected women (Boccia et al., 2016).

In Italy, the 2014–2018 National Prevention Plan, approved by the State-Regions Conference, called on the Italian regions to implement dedicated pathways well before the onset of breast cancer for high-risk women, by 2018. The experience of undergoing a diagnostic test of genetic mutation related to cancer risk gives the individual a greater possibility of choice over prevention on one hand, but deeply affects the thoughts, emotions, and beliefs about health and the perception of a possible disease, on the other hand.

Advances in prevention, treatment and increased survival of cancer patients results in the need for transformation of support and care. With genetic and scientific advances as well as new technologies, there is a need for a deep comprehension of the psychological effects of genetic diagnosis on individuals and their families. The introduction of the genetic test, BRCA 1/2, has allowed the identification of inherited abnormalities in genes that regulate the proper growth of cells in the breast and ovaries. Women carrying a BRCA1/2 mutation are at high risk of developing breast and/or ovarian cancer during their lifetime (Mavaddat et al., 2013).

BRCA genetic test results define “a particular emotional state related to lifestyle choices possible and to live with a perception of high risk of cancer” (Rania and Migliorini, 2015b, p. 100). If the experience of the encounter with cancer marks a profound turning point in the life of people that changes perspectives, time perception, emotions, relationships, and priorities, then more room for a greater emotional connection is required by meeting with medical quality personnel, psychologists, nurses, and physiotherapists. It is a being together, although in different positions, that can facilitate the task of reconstructing the puzzle of one’s illness and existence (Migliorini, 2015).

Once a person becomes aware of a risk and a probability of serious illness, he/she has to face a high level of uncertainty about their life and manage information and choices that genetic diagnosis involves. In fact, new technologies lead us to perceive ourselves according to the diagnoses that we receive before experiencing the associated disease condition (Rania and Migliorini, 2015b).

Having a mutation in BRCA1/2 genes leads to an experience of a state of constant uncertainty. These women are persistently living with the fear of receiving bad news: a cancer diagnosis. Woman with a positive BRCA test result diagnosis experience the challenges of managing a new self-perception, self-esteem, body-image and future health, leading to possible psychological distress as well as the interference with planning the future. While these women are not sick, they are no longer completely healthy. The new technologies create the need for the individual to internalize an idea of inevitable risk because they introduce the possibility to be in a condition where you have not yet been and where maybe you will never be (Duden, 2006).

Self-perception of people’s health is characterized by having positive feelings toward oneself, a feeling of self-control and an optimistic view of the future (Seligman and Csikszentmihalyi, 2000).

d’Agincourt-Canning (2006) argues that genetic information has generally been considered as *enabling* (authorized) as it allows subjects to participate in surveillance or undergo prophylactic surgery.

In predictive genetic diagnoses, paradoxically, the disease and the risk of contracting it become the same thing (Aronowitz, 2007). This condition makes very flexible the borders between the person with symptoms and the one without symptoms (Konrad, 2005; Nowotny and Testa, 2012). Furthermore, these genetic tests represent a challenge for these women not only from a medical point of view but also from a psychological one (Rania and Migliorini, 2015a). When looking for a way to reduce uncertainty about their level of risk by undergoing genetic testing, women who receive a positive test result are paradoxically occupied in a situation that on the one hand eliminates some worries but eventually adds new ones on the other hand (DiMillo et al., 2013). Within this condition, for some individuals, the illness and the risk of illness may become the same (Aronowitz, 2007). For example, “the result of genetic testing leads the individual to identify with the diagnosis of ‘his/her being so’, not something that ‘he/she feels’ or that ‘he/she owns’ but that ‘he/she is”’ (Rania and Migliorini, 2015a, p. 79).

The communication of risk within the genetic diagnosis introduces the person to the possibility of making decisions and making choices related to risk management. The literature refers to the goals of new technologies and to the complexity of our society, in which it is necessary to make difficult decisions in a short time (McEwen, 2011). Individuals use different strategies to address such decisions, some rational, such as weighting costs and benefits, calculating real risks, and others less rational, such as faith and trust, experiencing differentiated emotions.

The decision-making process presents itself as complex and characterized by ambivalence and should be supported by genetic counseling model that in recent years has been developing in order to provide effective decision-making support (Battistuzzi et al., 2019); among the decisions, a significant place is reserved for the choice of maternity and its possible anticipation within preventive prophylaxis (Quinn et al., 2010; Ormondroyd et al., 2012). According to Martinez et al. (2016) “women are increasingly interested in taking more active roles in their breast cancer treatment decisions” (p. 1950); but in this transition they encounter numerous complex decisions. A genetic counseling could facilitate shared decision making, it could help the patients to feel that they have the competence as well as the support to make medical decisions consistent with their values and preferences, satisfying autonomy and relatedness needs.

In this specific case study of health innovation, related to genetic testing, SDT could be a useful framework for understanding the integration process and the pursuit of autonomous goals to enhance individual well-being.

## Conclusion

In this paper, we tried to illustrate why health innovation has made some psychological processes related to the concept of health and disease more complex. The health innovation paradigm prefigures psychological challenges that were not previously considered. In particular, a psychological aspect that becomes salient is the possibility that new technologies allow people to be proactive and aware of their own psychological needs. Perceived autonomy is of great importance in every area of human behavior because it exerts an influence on the regulation of behavior through affections and cognitions, but it plays a central role in medical care. To cope with some specific choices concerning one’s own health, people can take an active role, satisfying the needs of competence, autonomy and relatedness. Within the framework of the biopsychosocial model, addressing the issue of self-determination becomes significant not only in the field of pathology but also in relation to physiological processes.

**FIGURE 2:**
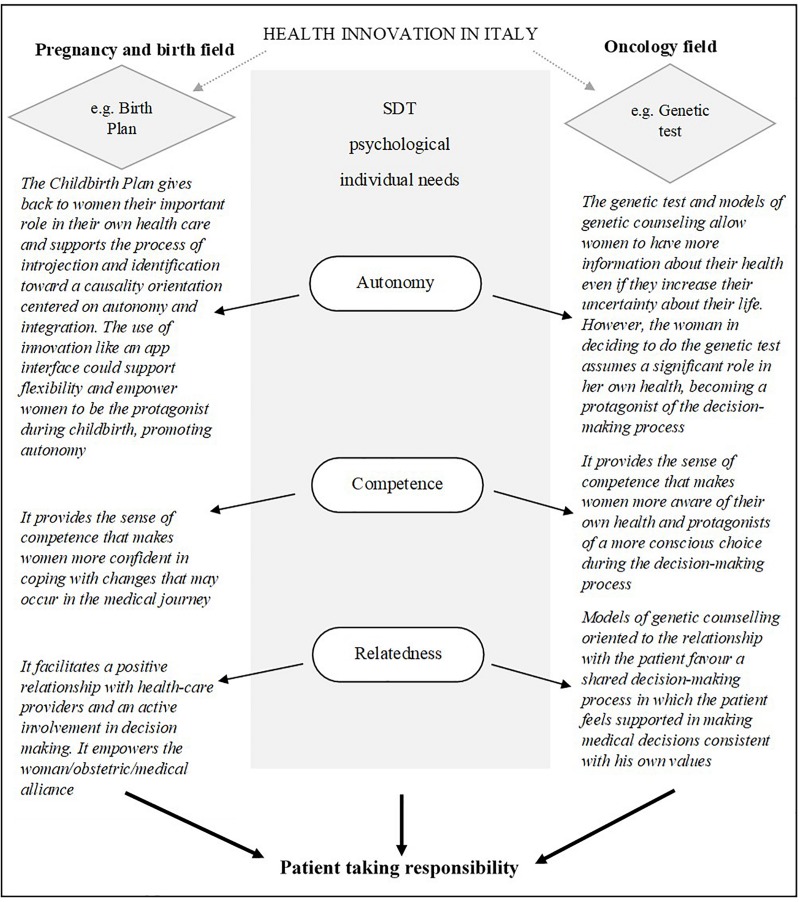
SDT as framework in physiological and pathological processes.

In the present paper, we chose two illustrative situations significant for the Italian context, the Childbirth Plan and the oncological genetic test, to highlight the possible role of SDT as a theoretical reference model (Figure 2). The process of decision making in choices concerning one’s own health in physiological and pathological contexts (e.g., delivery methods, treatment options) was the focus of our reflection and challenges us to think about how to build interventions based on the model proposed by SDT.

For researchers, it could be interesting to explore the relationship between SDT and the different stages of the process of patient care since, over time, it can change the emotional feeling and internal planning of the person (Houlis et al., 2018). In fact, even if SDT has already been operationalized, we hope for new research that can explore the potential for their utility in the context of the health innovation.

Practitioners could be stimulated to revise their orientation toward the patient and, according to SDT, they should support autonomy, rather than control, in promoting health-related choices to support patient well-being. A goal of professionals could be to enhance the individual resources involved in the decision-making process and the sense of self-determination of the person. This can develop a reflexive process aimed at enhancing one’s adaptability/flexibility. This ability appears fundamental within the processes activated by the practices introduced by health innovation that allow people to be informed and to plan possible implications for their health.

According to SDT, different situations could modify individuals’ functioning according to the psychological needs of recognition of competence, autonomy and relatedness. These needs become salient when the individual makes important health-related choices, as described above. The health innovation approach focuses on patients taking responsibility, as well as SDT. However, the risk inherent in this change of paradigm may be to go in the direction of reduced responsibility of the medical apparatus regarding the choice; our proposal, in accordance with SDT, is to activate a process of support for the person during the path, taking into account the individual’s potential and motivation. If patients feel they have little freedom, confidence and wellbeing in their lives and they are not supported in their decision making by healthcare professionals, as it sometimes occurs in VBAC decisions, they will hardly feel satisfied and their motivation will fail. Literature underlines that autonomy-supportive communication by healthcare professionals can improve patients’ perceived decision quality; this may be particularly important for breast cancer patients that experience a complex “emotional burden of anxiety, uncertainty, and fear” (Martinez et al., 2016, p. 1948). An additional risk could be to solicit in the person the illusion of a total control that can, rather than increase the well-being of the person, generate anxiety.

Healthcare professionals should keep these findings in mind when interacting with their patients if they have an interest in encouraging autonomy and well-being. Tools such as the Childbirth Plan can support people in decision making, providing the sense of competence that makes them more confident in coping with changes that may occur in the medical journey. In this direction, specific modules could be inserted into the birth preparation courses to support the self-determination of the woman, of the couple, and of the family. However, it seems very important that also medical educators pay greater attention to interpersonal relationship and communication in physician training to improve patients’ well-being (Williams and Deci, 1998; Williams et al., 2000).

In the pathological context, a psychological consultation, which takes into consideration the processes that the diagnosis can solicit, together with a genetic consultation, can help people to satisfy the needs of relatedness and to limit the sense of loneliness and disorientation in the face of the news.

Health care practitioners’ autonomy support encourages patients to boost their perceived competence in behaviors and can even enhance their sense of mindfulness (Martin et al., 2017). Our work could suggest possible practical implications for researchers and clinicians in order to improve tools and procedures to enhance listening to and addressing the needs and concerns of patients, supporting their sense of competence, autonomy and relatedness. Clinicians should create a need supportive climate, achieved by a combination of actions and communication styles (Ryan and Deci, 2017). However, literature underlines that individual techniques are not able to independently predict successful need support, reinforcing the suggestion that a need supportive environment requires a blending of multiple techniques (Gillison et al., 2019). The potential of SDT applied to health innovation is in the closeness between autonomy and relatedness needs, which are not contradictory positions, since the interpersonal dimension is not a limitation to individuality and self-realization but is, instead, a supportive element. A supportive climate, such as the one created by positive relationships in the medical context, could reduce the sense of threat in the individual and could therefore favor autonomous behavior. Through an active dialog with health professionals, the individual can acquire a greater mastery of the external environment and internalize experiences and values, thereby progressively developing an increasingly elaborate, adaptive and integrated sense of the self.

## Author Contributions

LM conceived the idea and was in charge of overall direction and planning. PC wrote the manuscript with support from NR. All authors discussed the conclusions and contributed to the final manuscript.

## Conflict of Interest Statement

The authors declare that the research was conducted in the absence of any commercial or financial relationships that could be construed as a potential conflict of interest.
